# Systemic Inflammatory Response Based on Neutrophil-to-Lymphocyte Ratio as a Prognostic Marker in Bladder Cancer

**DOI:** 10.1155/2016/8345286

**Published:** 2016-01-05

**Authors:** Hyung Suk Kim, Ja Hyeon Ku

**Affiliations:** Department of Urology, Seoul National University College of Medicine, Seoul 110-744, Republic of Korea

## Abstract

A growing body of evidence suggests that systemic inflammatory response (SIR) in the tumor microenvironment is closely related to poor oncologic outcomes in cancer patients. Over the past decade, several SIR-related hematological factors have been extensively investigated in an effort to risk-stratify cancer patients to improve treatment selection and to predict posttreatment survival outcomes in various types of cancers. In particular, one readily available marker of SIR is neutrophil-to-lymphocyte ratio (NLR), which can easily be measured on the basis of absolute neutrophils and absolute lymphocytes in a differential white blood cell count performed in the clinical setting. Many investigators have vigorously assessed NLR as a potential prognostic biomarker predicting pathological and survival outcomes in patients with urothelial carcinoma (UC) of the bladder. In this paper, we aim to present the prognostic role of NLR in patients with UC of the bladder through a thorough review of the literature.

## 1. Introduction

Cancer is a leading cause of morbidity and mortality presenting multifactorial features affected by a variety of factors, including tumor-related and host (patient)-related factors. Until recently, predicting outcomes in cancer patients have mainly depended upon tumor characteristics, such as pathologic tumor stage and tumor grade. However, various host-related factors, including weight loss (cachexia), performance status, and systemic inflammatory response (SIR), have been suggested as potential prognostic indicators in cancer patients.

Since Virchow first described a possible connection between inflammation and cancer in 1876 after observing the presence of leukocytes within neoplastic tissues [1], clear evidence now supports the crucial role played by SIR in the development, progression, metastasis, and survival of malignant cells in most cancers [2]. Most solid malignancies trigger an intrinsic inflammatory response that builds up a protumorigenic microenvironment. Inflammation in the tumor microenvironment may promote angiogenesis, invasion, and metastasis via the signaling of tumor-promoting chemokines and cytokines (i.e., IL-1, IL-6, tumor necrosis factor- [TNF-] a, and IL-23), which are produced by innate immune cells (macrophages, neutrophils, mast cells, myeloid-derived suppressor cells, dendritic cells, and natural killer cells) and adaptive ones (T and B lymphocytes) [1, 2]. Based on this background, in recent years many clinical studies have supported SIR as a meaningful predictor of survival outcomes in various types of cancers, including cancers of the lung [3–5], colorectum [6–11], gastrointestinal tract [12, 13], liver [14, 15], esophagus [16–18], breast [19–21], ovaries [22–24], cervix [25, 26], and pancreas [27]. In addition, the prognostic value of SIR has been vigorously assessed in urologic cancers, including prostate cancer [28–31], kidney cancer [32–34], and urothelial carcinoma (UC) (cancers of the bladder [35–48] and upper urinary tract (UUT) [49–60]).

UC is the second-most frequently diagnosed urologic malignancy. Clinical outcomes vary. A majority of UCs (90~95%) originate in the bladder, and UC of the UUT only accounts for 5~10% of all UCs. Radical cystectomy (RC) and radical nephroureterectomy (RNU), respectively, are applied as the gold standard local treatment for muscle-invasive or high-risk, non-muscle-invasive UC of the bladder and UUT. However, in spite of these aggressive local approaches, long-term prognosis remains poor due to disease recurrence accompanied by local and/or distant metastasis [61–63]. These poor outcomes suggest a need for ongoing risk stratification and proper selection of multimodal treatment approaches, such as chemotherapy in the neoadjuvant or adjuvant setting. To address these issues, a number of studies have explored SIR-related biomarkers as potential predictors of oncologic outcomes in UC. Among these, NLR, defined as the ratio of absolute neutrophils to absolute lymphocytes, has recently gained considerable attention as a biomarker in urothelial carcinoma (UC) arising from the bladder or upper urinary tract (UUT).

In this paper, we reviewed the clinical studies dealing with SIR-related biomarkers in association with oncologic outcomes in UC, with a special focus on NLR.

## 2. SIR-Based Prognostic Scoring System

Potential hematological biomarkers representing SIR in cancer patients include C-reactive protein (CRP), albumin, Glasgow Prognostic Score (GPS), modified GPS (mGPS), and neutrophil-to-lymphocyte ratio (NLR). The association of these SIR-related biomarkers with oncological outcomes has been extensively studied by many investigators in many types of nonurologic cancers (Table 1). Because hematological tests are routinely performed in most cancer patients, these biomarkers may be used as easily measurable, objective, reproducible, robust, and inexpensive parameters able to express the severity of SIR in cancer patients.

CRP is a nonspecific but sensitive marker of the acute phase response and is expressed in selected tumor cells [64]. The biological basis for the correlation between expression of this marker, cancer risk, outcome, and survival is not completely understood. Several proinflammatory cytokines, such as interleukin-1 (IL-1), IL-6, and TNF-a, expressed by the tumor environment induce CRP synthesis from the liver and other tissues [1, 2]. Based on many recent studies, it is now widely accepted that an elevated CRP value is a reliable indicator of poor prognosis in a variety of types of cancers [4, 8, 14, 16, 23, 28, 29, 31, 32, 65, 66].

Serum albumin, another marker of acute phase response to an inflammatory state, is generally used to assess nutritional status, severity of disease, disease progression, and prognosis [64]. Malnutrition and inflammation suppress albumin synthesis. In an adult, the normal range of serum albumin level is 3.5–5.0 g/dL. When levels drop below 3.5 g/dL, the condition is called hypoalbuminemia. The lower serum albumin concentration may be due to the production of cytokines such as IL-6, which modulate the production of albumin by hepatocytes [64]. Alternatively, TNF-a may increase the permeability of the microvasculature, thus allowing an increased transcapillary passage of albumin. Presence of micrometastatic tumor cells in the liver may induce the Kupffer cells to produce a variety of cytokines (IL-1, IL-6, and TNF-a), which may modulate albumin synthesis by hepatocytes [1, 2]. Thus, hypoalbuminemia is uncommon in early-stage cancer but as the disease progresses, albumin levels drop significantly and serve as good prognostic indicators in patients with various cancers [7, 19, 22, 67].

GPS and mGPS are inflammation-based prognostic scores developed by combining CRP and albumin to predict the clinical outcomes in cancer patients [68, 69]. GPS and mGPS, as routinely available, easily measured, and well standardized worldwide hematologic biomarkers, have subsequently been the subject of prognostic studies in wide variety of operable [13, 15, 18, 25, 27, 34] and inoperable [9, 10, 17, 20, 33] cancers. Indeed, these scoring systems have been extensively validated in various clinical scenarios and are now recognized to have prognostic value independent of tumor-based factors, such as pathologic tumor stage, tumor grade, lymphovascular invasion, and lymph node involvement.

It is also well recognized that SIR is related to changes in circulating white blood cells, especially an abnormal increase in neutrophils (neutrophilia) along with an abnormal decrease in lymphocytes (lymphocytopenia) [2, 64]. In light of this phenomenon under inflammatory conditions, NLR, being the ratio of neutrophils to lymphocytes, has gained considerable interest over the past decade not only as a potential prognostic factor associated with outcomes in a variety of cancers but also as a means of refining risk stratification of patients to treatment and predicting survival rates. Currently, NLR has been demonstrated to have significant prognostic value in urologic cancers, such as prostate [70] and renal cancer [71, 72], and also in cancers outside the urinary system [5, 11, 21, 24, 26].

## 3. SIR in Bladder Cancer

Prognosis in bladder cancer utilizes the same factors utilized for other types of cancers, including tumor-related factors, such as tumor stage, grade, lymphovascular invasion (LVI), and lymph node involvement (LNI) [61, 63]. However, all of these factors feature postoperative parameters. Given that SIR-related hematological biomarkers are easily obtained through pretreatment routine blood examination and have provided reliable prognostic information in other types of cancers, these biomarkers have been investigated in risk stratification for recurrence and mortality of patients with bladder cancer in both pre- and posttreatment settings. Several clinical studies have found an association between SIR-related hematological biomarkers, including CRP, albumin, and GPS, and oncologic outcomes of UC of the bladder (Table 3). In each different treatment setting, elevated CRP, defined as different cut-off (1.0 or 0.5 mg/dL),was significantly related to worse cancer-specific-survival (CSS) [35, 36]. One study demonstrated that in muscle-invasive bladder cancer (MIBC) patients with elevated CRP levels showed significantly more adverse pathologic features, such as extravesical disease (≥pT3), larger tumor size, lymph node involvement, and positive surgical margin prior to undergoing RC compared to patients with normal CRP levels. In addition, one-unit elevation in pre-RC CRP levels was significantly associated with a 20% increased risk of cancer-related death after RC [37]. In inoperable advanced bladder cancer, hypoalbuminemia and GPS 2 measured prior to chemotherapy were independently associated with shortened progression-free survival (PFS) and overall survival (OS), respectively [39]. Recently, Ku et al. developed a nomogram incorporating albumin, lymphocyte count, and platelet count to predict the probability of 5-year OS and disease-specific survival (DSS) after RC that demonstrated higher predictive accuracy than the existing staging system [46].

## 4. NLR in Non-Muscle-Invasive Bladder Cancer (NMIBC)

To date, few studies have assessed the association between NLR and the prognosis of NMIBC initially treated with transurethral resection of the bladder tumor (TURBT). Indeed, the evaluation of the prognostic role of NLR has been conducted with focus on MIBC patients undergoing RC or a mixed cohort of muscle-invasive and non-muscle-invasive tumors (Table 2). One recent study assessed the predictive value of preoperative NLR in 107 patients initially diagnosed with NMIBC following TURBT [47]. When applying each different cut-off point for NLR using the standardized cut-off finder algorithm, NLR > 2.41 and NLR > 2.43 were significantly associated with unfavorable disease progression and recurrence. Owing to the limited sample size of this study, further studies will be required to validate the role of NLR as a predictor for recurrence and progression in NMIBC.

## 5. NLR in Muscle-Invasive Bladder Cancer (MIBC)

In the past five years, the prognostic role of NLR in MIBC has been actively investigated in association with various oncological outcomes, including pathologic outcome, post-RC recurrence, and survival (Table 2). Several studies evaluated the association between NLR and post-RC survival outcomes [38, 40, 41, 44]. The cut-off point chosen to define an elevated NLR differed across studies, ranging from 2.5 to 3. Although one study reported no significant association between elevated NLR and OS [40], elevated NLR has been regarded as an independent predictor of RFS (recurrence-free survival), OS, and CSS in most studies [38, 41, 44]. One study reported that higher NLR values were observed in MIBC patients compared with NMIBC patients [42]. In addition, several studies demonstrated a significant correlation between a higher NLR and adverse pathologic outcomes, such as larger tumor size, pathological upstaging to locally advanced disease (pT3), and LNI after RC [41–44]. In locally advanced MIBC treated with neoadjuvant chemotherapy (NACH) prior to RC, continuous NLR decrease from before NACH to before RC was observed only in patients showing a pathological response after RC; therefore, sustained NLR decrease during NACH was suggested as a potential surrogate marker reflecting the effect of NACH [48]. The aforementioned studies mainly dealt with the prognostic value of NLR in the pretreatment setting. Interestingly, one recent study elucidated the influence of posttreatment NLR measured in the early post-RC period on oncologic outcomes [45]. The cut-off point of pre- and post-RC NLR (2.1 and 2.0, resp.) was differently determined according to each receiver operating characteristics (ROC) curve analysis. Similar to the aforementioned study results, elevated NLR after RC was also significantly associated with adverse pathologic outcomes, such as pT3/T4 disease, LVI, and LNI, and was an independent predictor of OS and CSS. Moreover, patients with perioperative continuous elevated NLR (2.1 ->2.0) showed worse OS and CSS compared with other change groups. Therefore, pre- and posttreatment NLR might have prognostic value in predicting postoperative survival outcome in patients with MIBC.

## 6. NLR in Upper Urinary Tract Urothelial Carcinoma (UTUC)

Similar to bladder cancer, the prognostic significance of other SIR-related hematological biomarkers, including CRP, albumin, and neutrophil count, has been proven to be reliable in terms of predicting adverse pathologic and survival outcomes following definitive surgery in UTUC [49, 51, 52, 55, 58]. In recent years, the prognostic role of NLR has also been vigorously assessed in UTUC [50, 53, 56, 57, 60] (Table 3). Although all of the studies involved cohorts of patients with operable UTUC, the threshold to determine elevated NLR levels was not uniform, ranging from 2.5 to 3. However, irrespective of the choice of NLR threshold, elevated NLR over the threshold was consistently correlated with adverse postoperative pathologic findings (high tumor grade, advanced tumor stage, LVI, and LNI) and worse survival outcomes following RNU.

## 7. Clinical Implications of SIR in Bladder Cancer

NMIBC can primarily be treated with TURBT. However, frequent recurrence (50~70%) and progression (10~20%) rates after TURBT are a major concern [61, 73]. Management of NMIBC might involve lifelong surveillance and place a considerable economic burden on patients. Currently, cystoscopy is the standard of care during the surveillance period. It is, however, invasive, and repeated cystoscopic examinations can cause substantial discomfort and pain to patients. Although investigators have developed various models to predict recurrence and progression after TURBT for NMIBC including nomogram, scoring systems, and risk tables [74–77], these models mainly incorporated tumor-related factors, such as tumors number, tumor diameter, T category, World Health Organization (WHO) tumor grade, and carcinoma in situ (CIS). Considering the significant correlation of elevated NLR with disease recurrence and progression in NMIBC [47], the addition of NLR to the existing prediction model may contribute to more accurate stratification of patients with NMIBC according to risk of recurrence and progression. Also, according to risk stratification based on pretreatment NLR values, selective cystoscopic examination and additional treatment, including intravesical Bacillus Calmette-Guérin (BCG) immunotherapy or chemotherapy, will be possible in patients with high-risk NMIBC, thereby reducing their economic burden and the potential discomfort caused by repeated cystoscopy.

In terms of MIBC, one significant challenge has been the limited, pretreatment, risk-stratification data that exists for patients undergoing RC. The well-established risk factors for recurrence and survival in MIBC included tumor-related factors, including pathologic tumor stage, pathologic tumor grade, CIS, LVI, and LNI [78–80]. Moreover, most predictive models (nomogram) predicting recurrence and survival in bladder cancer have been heavily based on postoperative pathologic factors, such as pathologic tumor stage, pathological grade, LVI, and LNI [81–83], with minimal consideration for associated host-related factors. Meanwhile, the accuracy of clinical staging in bladder cancer remains poor, reporting upstaging rate of 50% at RC specimen [84]. Thus, not enough data exists to facilitate appropriate patient counseling and guide clinical trial enrollment. As such, it is required to identify biomarkers that can assist with preoperative patient risk stratification and counseling. To achieve these goals, SIR-related hematological biomarkers can be a potential and promising factor. Assessment of SIR-related biomarkers in bladder cancer may be particularly relevant, because the inflammatory process seems to play an important role in the genesis and progression of, as well as mortality from, bladder cancer [1, 2]. Based on the previous study result [43], demonstrating a significant association between pretreatment elevated NLR and pathologic upstaging after RC, the performance of early cystectomy or NACH prior to RC might be considered in patients with pretreatment high NLR to attain tumor downstaging and improve postoperative survival. In addition, the pattern of change in NLR during NACH will be a valuable surrogate marker for monitoring and predicting pathological response to NACH [48]. Several studies reported the incorporation of SIR-related hematological biomarkers, such as CRP, NLR, albumin, and lymphocyte and platelet count, with a predictive model for survival outcomes in MIBC [37, 38, 46] or UTUC [50, 55, 57], improved predictive accuracy of the model, and consequently discriminated patients well according to risk stratification. It follows that pretreatment evaluation of NLR will be helpful in counseling patients about their prognosis.

A recent study revealed that the NLR value measured during the early postoperative period (from 1 to 3 months) after RC had a significant correlation with adverse oncological and survival outcomes [45]. Thus, postoperative NLR and the pattern of NLR change in the perioperative period may also provide valuable information in determining which patients should be referred for additional multimodal treatment, such as radiation and adjuvant chemotherapy.

The limitations of current NLR-associated studies in cancer are as follows. First, as mentioned earlier, there was no uniform cut-off point for NLR; each threshold was adopted according to a variety of statistical methodologies. Unlike tumor-related prognostic factors, including pathologic tumor stage and grade, NLR as a host-related factor can be affected by a variety of physiologic conditions, such as patients' comorbidities (hypertension and diabetes mellitus) and type of cancer, which can trigger immune response to cancer so that the establishment of definite NLR threshold may be difficult in consideration of these changeable physiologic conditions among cancer patients. Second, nearly all of the studies were both clinical and retrospective. Further large-scale prospective clinical or experimental animal (preclinical) research using a unified and robust statistical methodology will be required to determine the definite cut-off value of NLR and to discover the biological mechanisms supporting the correlation between NLR and oncologic outcomes in cancer patients.

## 8. Conclusion

Elevated NLR has shown a significant association with adverse oncologic and survival outcomes in patients with UC. Thus, NLR as a potential marker of SIR may become a promising tool in the management of patients with UC of the bladder and UUT, in terms of improved risk assessment for prognosis and guidance for treatment. Moreover, the ease and convenience of routine blood examinations in the clinical setting mean that NLR can be an objective, inexpensive, reproducible, and cost-effective measurement for the prediction of prognosis in UC. However, current NLR-related studies have not applied uniform NLR thresholds and thus require cautious interpretation because of many statistical methodological limitations. For the introduction of NLR into the clinical practice, rigorous attempts should be made in proper prospective study design.

## Figures and Tables

**Table 1:** Clinical studies on the prognostic value of SIR-related hematological biomarkers in various types of cancers other than UC.

Study	Marker	Type of cancer	Threshold	Assessment period	Results
Parker et al. [22]	Albumin	Ovarian cancer	3.5 & 4.1 g/dL	Before operation	Low-albumin level (continuous value) was associated with worse OS

Lis et al. [19]	Albumin	Breast cancer	3.5 g/dL	Before operation	Low-albumin level (<3.5 g/dL) was related to higher death rate

Lai et al. [7]	Albumin	Colon cancer	3.5 g/dL	Before operation	Hypoalbuminemia (<3.5 g/dL) was associated with increased morbidity and mortality

Seebacher et al. [67]	Albumin	Endometrial cancer	4.21 g/dL or continuous	Before operation	Increased albumin level (continuous) was related to better DFS and PFS

Hashimoto et al. [14]	CRP	HCC	1.0 mg/dL	Before operation	Elevated CRP (>1) was significant predictor of worse OS and RFS

Lehrer et al. [28]	CRP	Prostate cancer	NA (continuous)	Before radiation	There was a significant correlation of CRP level with PSA

Crumley et al. [16]	CRP	Gastroesophageal cancer	1.0 mg/dL	Before operation	Elevated CRP (>1) was independent predictor of CSS

Jones et al. [4]	CRP	Lung cancer	0.4 mg/dL	Before operation	Elevated CRP (>0.4) was related to larger tumor size, advanced tumor stage, and incomplete resection

Karakiewicz et al. [32]	CRP	RCC	0.4 & 2.3 mg/dL	Before nephrectomy	Elevated CRP (>2.3) was an informative predictor of worse CSS

Beer et al. [29]	CRP	Metastatic prostate cancer	0.8 mg/dL	Before docetaxel based chemotherapy	Elevated CRP (>0.8) was a strong predictor of poor OS and lower PSA response to chemotherapy

Hefler et al. [23]	CRP	Ovarian cancer	1.0 mg/dL	Before surgery	Elevated CRP (>1.0 & continuous) was associated with postoperative residual tumor and worse OS

Shiu et al. [8]	CRP	Colorectal cancer	0.5 mg/dL	Before surgery	Elevated CRP (>0.5) was correlated with larger tumor size, higher stage, and poorer CSS

Crumley et al. [17]	GPS	Inoperable gastroesophageal cancer	1.0 mg/dL (CRP) 3.5 g/dL (Albumin)	Before nonsurgical treatment	High GPS was significant predictor of worse CSS

Al Murri et al. [20]	GPS	Metastatic breast cancer	1.0 mg/dL (CRP) 3.5 g/dL (Albumin)	Before non-surgical treatment	High GPS was significant predictor of worse CSS

Ramsey et al. [33]	GPS	Metastatic RCC	1.0 mg/dL (CRP) 3.5 g/dL (Albumin)	Before treatment	High GPS was significant predictor of worse CSS

Polterauer et al. [25]	GPS	Cervical cancer	1.0 mg/dL (CRP) 3.5 g/dL (Albumin)	Before surgery	High GPS was significant predictor of worse OS and DFS

Vashist et al. [18]	GPS	Esophageal cancer	1.0 mg/dL (CRP) 3.5 g/dL (Albumin)	Before surgery	High GPS was a strong prognosticator of perioperative morbidity and worse DFS and OS

Kinoshita et al. [15]	GPS	HCC	1.0 mg/dL (CRP) 3.5 g/dL (Albumin)	Before treatment	High GPS was independently associated with worse CSS

Leitch et al. [9]	mGPS	Colorectal cancer (operable or unresectable)	1.0 mg/dL (CRP) 3.5 g/dL (Albumin)	Before treatment	High mGPS was independently associated with worse CSS in patients with either operable or unresectable colorectal cancer

Jiang et al. [13]	mGPS	Gastric cancer	1.0 mg/dL (CRP) 3.5 g/dL (Albumin)	Before surgery	High mGPS was independently associated with worse OS irrespective of cancer stage

Ishizuka et al. [10]	mGPS	Unresectable colorectal cancer	1.0 mg/dL (CRP) 3.5 g/dL (Albumin)	Before chemotherapy	High mGPS (1/2) was an independent risk factor of poor CSS

La Torre et al. [27]	mGPS	Pancreatic cancer	1.0 mg/dL (CRP) 3.5 g/dL (Albumin)	Before surgery	High mGPS was independently associated with worse OS irrespective of cancer stage

Lamb et al. [34]	mGPS	RCC	1.0 mg/dL (CRP) 3.5 g/dL (Albumin)	Before surgery	High mGPS was significantly independent predictors of worse OS and CSS

Cho et al. [24]	NLR	Ovarian cancer	2.6	Before surgery	Positive NLR (>2.6) showed worse OS and DFS than negative NLR (<2.6)

Chua et al. [11]	NLR	Metastatic colorectal cancer	5	Before chemotherapy	Elevated NLR (>5) was independently associated with less clinical response to chemotherapy and worse OS and PFS

Azab et al. [21]	NLR	Breast cancer	Multiple cut-offs (1.8, 2, 45, 3, 33)	Before chemotherapy	High NLR (>3.3) was an independent significant predictor of all-cause mortality

Keizman et al. [70]	NLR	Metastatic CRPC	3	Before ketoconazole	Low NLR (≤3.0) was significantly associated with better PFS

Keizman et al. [71]	NLR	Metastatic RCC	3	Before sunitinib	Low NLR (≤3.0) was independent predictor of better response to sunitinib and favorable PFS and OS

Lee et al. [26]	NLR	Cervical cancer	1.9	Before treatment	High NLR (≥1.9) was related to more advanced stage and increased NLR (continuous) was an independent predictor of worse PFS and OS

Yao et al. [5]	NLR	Advanced lung cancer	2.63	Before chemotherapy	Low NLR (≤2.63) was independently associated with better clinical response to chemotherapy and favorable OS and PFS

RCC: renal cell carcinoma, HCC: hepatocellular carcinoma, CRP: C-reactive protein, GPS: Glasgow Prognostic Score, mGPS: modified Glasgow Prognostic Score, NLR: neutrophil-to-lymphocyte ratio, OS: overall survival, DFS: disease-free survival, PFS: progression-free survival, and CSS: cancer specific survival.

**Table 2:** Clinical studies on the prognostic value of SIR-related hematological biomarkers in UC of the bladder.

Study	Marker	Publication year	Number of patients (NMIBC/MIBC)	Threshold	Assessment period	Main findings
Hilmy et al. [35]	CRP	2005	105 (76/29)	1.0 mg/dL	Before surgery (TURBT)	Elevated preoperative CRP (>1) was independently associated with worse CSS

Yoshida et al. [36]	CRP	2008	88 (0/88)	0.5 mg/dL	Before radiochemotherapy	Elevated preoperative CRP (≥0.5) was independent predictor of worse CSS

Gakis et al. [37]	CRP	2011	246 (0/246)	0.5 mg/dL or continuous	1–3 days before RC	Patients with elevated CRP (>0.5) showed advanced age, more extravesical disease, larger tumor size, node positive disease, and positive surgical margin and increased CRP (continuous) was independent predictor of worse CSS

Hwang et al. [39]	GPS, Albumin	2012	67 (0/67)	1.0 mg/dL (CRP) 3.5 g/dL (Albumin)	1 day before first chemotherapy cycle	Hypoalbuminemia (<3.5) and GPS 2 was independently associated with reduced PFS and OS, respectively

Ku et al. [46]	Albumin Neutrophil count Platelet count	2015	419 (173/246)	3.5 g/dL (Albumin) 7500/uL (Neutrophil) 400 × 10^5^/ul (Platelet)	Before RC	Low albumin, high lymphocyte count, and high platelet count were significantly associated with worse OS and CSS

Gondo et al. [38]	NLR	2012	189 (62/127)	2.5	Before RC	Elevated NLR (≥2.5) was an independent predictor of worse DSS

Demirtaş et al. [40]	NLR	2013	201 (35/166)	2.5	Before RC	Elevated NLR (>2.5) was not associated with overall survival

Hermanns et al. [41]	NLR	2014	424	3	Before RC	Patients with elevated NLR (≥3) significantly showed more advanced pathologic tumor stage Elevated NLR (≥3) was significantly associated with RFS, OS, and CSS

Kaynar et al. [42]	NLR	2014	291 (192/99)	NA (continuous)	1 day before surgery (TURBT or RC)	Patients with MIBC showed significantly higher NLR value than those with NMIBC Also, higher NLR significantly correlated with advanced age, larger tumor size, and aggressive tumor invasiveness

Potretzke et al. [43]	NLR	2014	102 (31/71)	NA (continuous)	Before RC	NLR was significant predictor of pathological upstaging after RC; also, patients with pathological upstaging to ≥pT3 had a significantly greater NLR compared to patents who remained at ≤pT2

Viers et al. [44]	NLR	2014	899 (392/507)	2.7	Within 90 days before RC	Elevated NLR (≥2.7) was significantly associated with adverse pathologic finding (higher pathologic tumor stage, node positive, and larger tumor size); increased NLR (continuous) was independently associated with worse RFS, OS, and CSS

Mano et al. [47]	NLR	2015	107 (107/0)	2.41 (for progression) 2.43 (for recurrence)	Before TURBT	Elevated NLR (>2.41) showed more pT1 tumors and was significantly associated with disease progression; elevated NLR (>2.43) was independent predictor of disease recurrence

Seah et al. [48]	NLR	2015	26 (0/26)	NA	Before NACH, during NACH, and after RC	Significant NLR decrease from before NACH to before RC was observed in patients with pathological response after NACH and RC

Kang et al. [45]	NLR	2015	385	2.0 (postoperative) 2.1 (preoperative)	Within 1 month before RC and within 3 months after RC	Patients with elevated postoperative NLR (≥2.0) had higher rates of ≥pT3, LVI, and positive lymph node and elevated postoperative NLR (≥2.0) was an independent predictor of OS and CSS; also, patients with perioperative continuous elevated NLR (2.1–>2.0) showed worse OS and CSS compared with other change groups

CRP: C-reactive protein, GPS: Glasgow Prognostic Score, mGPS: modified Glasgow Prognostic Score, NLR: neutrophil-to-lymphocyte ratio, TURBT: transurethral resection of bladder tumor, RC: radical cystectomy, NACH: neoadjuvant chemotherapy, NMIBC: nonmuscle invasive bladder cancer, OS: overall survival, DSS: disease-specific survival, RFS: recurrence-free survival, and CSS: cancer specific survival.

**Table 3:** Clinical studies on the prognostic value of SIR-related hematological biomarkers in upper urinary tract urothelial carcinoma.

Study	Marker	Publication year	Number of patients	Threshold	Assessment period	Main findings
Saito et al. [49]	CRP	2007	130	0.5 mg/dL	Before surgery	Patients with elevated (>0.5) CRP showed higher hemoglobin, advanced tumor stage (≥pT3), positive lymph node, high grade, and LVI; moreover, elevated (>0.5) CRP was significant prognostic factor for DSS and RFS

Obata et al. [52]	CRP	2013	183	0.5 mg/dL	Before surgery	Patients with elevated (>0.5) CRP showed advanced tumor stage (≥pT3), LVI, and higher number of metastases; moreover, elevated (>0.5) CRP was significant prognostic factor for worse RFS and CSS

Tanaka et al. [58]	CRP	2014	564	Multiple cut-offs (0.5, 2.0 mg/dL)	Before surgery	Elevated CRP (0.5–2.0 or >2.0) level was an independent predictor of worse RFS and CSS relative to normal CRP (≤0.5); in elevated pre-CRP (>0.5) group, postoperative normalization of CRP (≤0.5) was an independent predictor of better CSS

Ku et al. [55]	Albumin	2014	181	3.5 g/dL	Before surgery	Hypoalbuminemia (<3.5) was a significant predictor of worse DSS and OS; also, scoring model incorporated albumin discriminated patients well according to risk of DSS and OS

Hashimoto et al. [51]	Neutrophil count	2013	84	4000/uL	Before surgery	Elevated neutrophil count (≥4000/uL) was an independent prognostic factor for worse RFS

Azuma et al. [50]	NLR	2013	137	2.5	Before surgery	Elevated (≥2.5) NLR was significantly associated with worse RFS and CSS; also, scoring model incorporated NLR discriminated patients well according to risk of RFS and CSS

Dalpiaz et al. [53]	NLR	2014	202	2.7	Before surgery	Elevated (≥2.7) NLR was significantly associated with worse OS and CSS

Luo et al. [56]	NLR	2014	234	3	Before surgery	Elevated (≥3) NLR was significantly associated with worse MFS and CSS; also, the use of a NLR of >3 further identified a poor prognostic group, especially in patients with pT3 for MFS and CSS

Tanaka et al. [57]	NLR	2014	665	3	Before surgery	Patients with elevated (>3) NLR significantly showed high tumor grade (Gr 3), advanced tumor stage, positive lymph node, and LVI; elevated (≥3) NLR was an independent risk factor for worse RFS and CSS; furthermore, addition of pre-NLR slightly improved the accuracies of the base model for predicting both RFS and CSS

Sung et al. [60]	NLR	2015	410	2.5	Before surgery	Elevated NLR (≥2.5) was independent predictor of worse PFS, OS, and CSS, along with elevated ESR

CRP: C-reactive protein, NLR: neutrophil-to-lymphocyte ratio, LVI: lymphovascular invasion, OS: overall survival, DSS: disease-specific survival, RFS: recurrence-free survival, MFS: metastasis-free survival, and CSS: cancer specific survival.
